# Cultural Safety Knowledge and Practices Among Internationally Qualified Nurses Caring for Indigenous Peoples in Australia, New Zealand and Canada: A Scoping Review

**DOI:** 10.1177/10436596251353518

**Published:** 2025-07-22

**Authors:** Pratibha Bhandari, Ling Zeng, Anne-Marie Eades, Danielle Manton, Annie Hepworth, Carolyn Antoniou, Elaine Correia Moll, Jack Cornish, Suzanne Sheppard-Law

**Affiliations:** 1 University of Technology Sydney, New South Wales, Australia; 2 Curtin University, Perth, Western Australia, Australia; 3 The Prince of Wales Hospital, Randwick, New South Wales, Australia; 4 Sydney Hospital & Sydney Eye Hospital, New South Wales, Australia

**Keywords:** cultural safety, cultural capability, Indigenous peoples, internationally qualified nurses, registered nurses

## Abstract

**Introduction:**

Culturally safe practices are crucial for equitable health care for Indigenous Peoples. Despite the vital role of internationally qualified nurses in delivering patient care in the host countries, there is limited evidence on their knowledge and practices of cultural safety. This paper, aims to identify and map existing evidence on cultural safety knowledge and practices among internationally qualified nurses in Australia, New Zealand and Canada.

**Method:**

A scoping review was conducted using a comprehensive search strategy across five electronic databases and gray literature.

**Results:**

Three studies met the inclusion criteria. Findings were grouped into two categories: knowledge on cultural safety and challenges in translating this knowledge into practice.

**Discussion:**

Our review highlights the scarcity of evidence in this area. The findings from the limited existing evidence underscore the urgent need to plan future research on knowledge and practices related to cultural safety among internationally qualified nurses to promote health outcomes for Indigenous Peoples.

## Introduction

Registered nurses (RN) constitute the largest cohort of registered health care professionals globally, accounting for approximately 59% of the total health care workforce (World Health Organisation [WHO], 2020). In many Western countries, a substantial portion of the registered nurse workforce were trained and qualified overseas (OECD, 2019).

An internationally qualified nurse (IQN) is defined as an individual who has completed their undergraduate nursing education program from an educational institution located outside of the host country. While the acronym IQN is commonly used in Australia and New Zealand, the term internationally educated nurses (IEN) may be more typical in Canada and the United States. In this paper, the term IQN will be consistently used to refer to this critical population of nurses found across health care systems globally.

The Australian Health Practitioner Regulation Agency (AHPRA) reported that 32.3% (*n* = 11,188) of new registrations granted in 2022–2023 were for IQNs (AHPRA, 2023). Similarly, in Canada, IQNs comprised 64% of the net increase in RN registrations in 2022 (Canadian Institute for Health Information, 2024). While IQNs bring diverse skill sets and perspectives that help address workforce shortages; they often face challenges in caring for diverse populations and may not be familiar with all cultural nuances and health care practices in their host countries.

Indigenous Peoples worldwide have faced discrimination, disruption, and displacement due to colonization. The effects of this historical and intergenerational trauma have led to persistent inequities in health care, continuing to impact the physical and mental health outcomes of Indigenous Peoples globally (Gone et al., 2019; McGough et al., 2022). Indigenous Peoples from different regions share common experiences of displacement, loss of land, and autonomy, leading to cultural barriers and distrust of the mainstream health system, however despite these commonalities, the experiences of each group are unique and cannot be generalized. Nevertheless, country-specific policies and the structure of health care systems commonly affect access and experiences for Indigenous People. Due to similarities in the health care systems, this review includes studies conducted among Aboriginal and Torres Strait Islander Peoples from Australia, First Nations Peoples from Canada, and Māori from New Zealand. For consistency, we have used the term Indigenous Peoples throughout this study.

Cultural competence is a broad concept that has been emphasized as an essential skill for providing inclusive care to people from diverse backgrounds and cultures (Sharifi et al., 2019). While cultural competence focuses on the capacity of nurses to deliver care, it falls short of promoting health equity among Indigenous Peoples as it does not incorporate self-reflection on power dynamics and fails to prioritize the experiences and perspectives of patients receiving care (Curtis et al., 2019). On the other hand, cultural safety refers to the practice of creating an environment in which individuals feel respected and safe (Public Health Agency of Canada, 2023). Cultural safety requires health care providers to examine their own cultural identities and attitudes and strive to address the power imbalance inherent in the health care system (Curtis et al., 2019). Health care attitudes and power imbalances are experienced by Indigenous Peoples as racism, discrimination, and intergenerational trauma which contribute to inequity and poor health outcomes. Cultural safety is an essential component of nursing practice which requires nurses to advance the rights of Indigenous Peoples, to access health care services without any discrimination (Australian Human Rights Commission, n.d.). Cultural safety is a collaborative and inclusive approach that acknowledges the power dynamics in health care interactions and respects and addresses the unique care needs of Indigenous Peoples to promote positive health outcomes (Tremblay et al., 2023). Inclusive practices represent a shift from a one-size-fits-all approach to one that includes cultural awareness and cultural respect (McGough et al., 2022). Culturally safe practices require nurses to adopt a person-centered approach and work collaboratively with Indigenous Peoples, families and communities, as cultural safety can only be determined by Indigenous Peoples, families and communities (Australian Health Practitioner Regulation Agency, 2020). There is evidence that culturally unsafe practice by health care professionals can decrease Indigenous peoples’ access to health care service and engagement in health management, resulting in poor health outcomes (McGough et al., 2022) while culturally safe practices that are responsive to Indigenous peoples’ beliefs and values result in improved health outcomes (Curtis et al., 2019; McGough et al., 2022).

Access to culturally safe health care is a key factor in reducing health inequity and aligns with human rights principles; however, its translation to practice requires knowledge of historical and contemporary contexts, as well as relevant skills and strategies (Cox & Best, 2022). To provide culturally safe care, nurses are required to develop an understanding of Indigenous Peoples’ culture, language and colonization history. This knowledge enables nurses to examine their own personal attitudes, values and beliefs, and foster cultural humility (Canadian Institute for Health Information, 2021; Nursing and Midwifery Board AHPRA, 2023; Te Tāhū Hauora Health Quality & Safety Commission, 2021).

Nurses trained in Australia, Canada, and New Zealand have cultural safety education embedded in their nursing curricula mandated by nursing accrediting bodies in all three countries. The education is culturally specific to the host country and designed to embed relevant knowledge and awareness of cultural safety, to ensure the cultural capabilities across all health care. IQNs play a vital role in delivering patient care however their knowledge of cultural capabilities specific to the host country may be limited compared with nurses trained in the host country, primarily due to unfamiliarity with Indigenous history and culture. They may also be less aware of the practices related to the development of culturally safe care, such as continuous engagement in self-reflection on their personal worldviews, potential biases, and power imbalances that could compromise patient cultural safety (Cox & Best, 2022). Opportunities for IQNs to develop these practices to support their understanding, practice, and attitudes related to cultural safety remain unclear. This scoping review aims to identify and map the available evidence regarding knowledge and practices related to cultural safety among IQNs working in health care organizations across Australia, New Zealand, and Canada.

### Review Question

This review question was as follows:

What is the level of knowledge and practices on cultural safety among the IQNs working in health care organizations across Australia, New Zealand and Canada?

## Method

### Design

Given that little is known about the knowledge and practices of IQNs on cultural safety, the methodology of a scoping review was chosen to map the breadth of the literature and identify the characteristics in this field, including a variety of methodological designs and sources of literature (Peters et al., 2020; Pollock et al., 2021; Tricco et al., 2016). The review process was guided by the Joanna Briggs Institute (JBI) methodology for the conduct of scoping reviews (Peters et al., 2020). The systematic process of the review was reported in line with the Preferred Reporting Items for Systematic Reviews and Meta-Analyses Extension for Scoping Reviews (PRISMA-ScR) checklist (Tricco et al., 2018).

### Inclusion and Exclusion Criteria

#### Participants

RN, enrolled nurses, and nurse practitioners who are registered and currently practising in Australia, New Zealand, and Canada were included in our review. IQNs were defined as nurses whose primary nursing qualification was completed outside of the host country. Midwifery knowledge and practice differ from nursing, and therefore, studies that focused exclusively on registered midwives were excluded. Studies related to IQNs caring for Indigenous Peoples in other countries such as the United States were excluded from this review as the aim was to compare countries with structurally similar health care systems and potentially comparable workforce makeup. In this study, we have used the term IQN, which includes all IQNs and IENs working in Australia, Canada, and New Zealand.

#### Concept

The concepts of interest for this scoping review were cultural capability and cultural safety. The review looked to include studies exploring or describing the knowledge, attitudes, awareness, practices or/and experiences of IQNs of cultural capability and cultural safety when providing health care to Indigenous Peoples. In this review, cultural safety was specifically referred to culturally safe practices delivered by IQNs in response to Indigenous Peoples’ beliefs, values, and culture. Studies reporting cultural safety regarding caring for host populations and culturally and linguistically diverse populations were excluded.

#### Context

Due to similarities in the historical context of colonization and the health care system, studies conducted in Australia, Canada, and New Zealand were included in this review. Studies reporting on cultural diversity were excluded from this review.

#### Sources of Evidence

This scoping review included all types of quantitative, qualitative, and mixed-methods studies to ensure the inclusion of all relevant literature. Gray literature, such as dissertations, Google searches, government documents and organizational guidelines, which discussed cultural safety education, policies and practices for IQNs were searched for this review. Articles that were not original research, such as expert commentary or conference abstracts, or where full text was not accessible, were excluded.

### Search Strategy

After identifying the review aim, a comprehensive computer-assisted search strategy was developed by combining key terms and Medical Subject Headings (MeSH) with the assistance of a librarian (see Table 1). Five electronic databases were targeted for searching: MEDLINE (Ovid), EMBASE, Cumulative Index to Nursing and Allied Health Literature (CINAHL), Web of Science and Scopus, published from inception to the last search in July 2024 in English (Supplementary Table 1). Dissertation, Google search, government documents and organizational guidelines and reference lists of included studies and relevant review papers were manually searched to maximize the number of eligible primary sources and reduce the search bias (Bradbury-Jones et al., 2022).

**Table 1.** Search Terms.

Concept	Internationally qualified nurses	AND	Cultural safety	AND	Country
Searching key terms	“Internationally qualified nurs*” OR “Overseas qualified nurs*” OR “Internationally trained nurs*” OR “Overseas trained nurs*” OR “Internationally educated nurs*” OR “IEN” OR “International nurs*” OR “Migrant nurs*” OR “Foreign-educated nurs*”		cultural*		Australia* OR Canad* OR “New Zealand”
Searching MeSH terms	Nurses, international/ foreign nurse/		Cultural competency/ Culturally competent care/ Cultural diversity/ Clinical competence/ Transcultural nursing/		

### Screening and Selection

**Figure 1.**
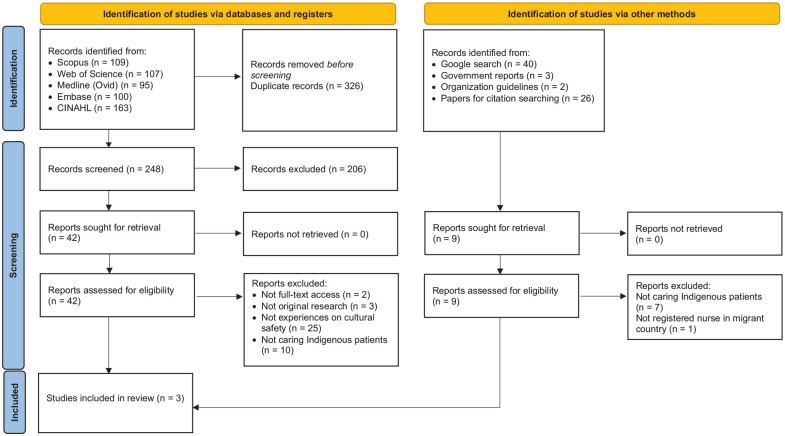
PRISMA Flowchart.

A total of 574 studies were initially identified and imported into EndNote 21 for managing and screening the search output. The review process is reported in the PRISMA chart (Figure 1).

Full text studies were imported into the software package Covidence for screening. Two reviewers independently assessed eligibility per inclusion and exclusion criteria. In cases of disagreement, a third reviewer was consulted until a consensus was reached.

### Data Extraction

Data from each paper was extracted and tabulated in Table 2, encompassing the first author’s name of publication, year of publication, data resource, country for the study, study objective, study designs and setting, participants and key findings for addressing the review question. Two reviewers independently extracted the data and checked repeatedly to ensure no key findings were missed. Any disagreement was discussed among the research team until the discrepancies were resolved.

**Table 2.** Data Extraction Table.

Author (Year)	Data type	Country	Study objectives	Study design & setting	Participants	Key findings
C. E. Z. Baxter (2017)	Doctoral dissertation	Canada	To explore and describe the integration of internationally educated nurses from the Philippines into the current nursing workforce in Canada	Mixed methods study (Online survey & a qualitative descriptive interview)	Internationally educated registered nurses from the Philippines who were currently working as Licensed practical nurse (LPN) (*n* = 12) and Registered Nurses (*n* = 10) in Canada	• Formal support provided by employers, including from the pre-licensure stage was considered valuable in helping the internationally educated nurses to understand country specific health care system and culturally safe practices, especially the First Nation’s culture.
M. Brunton et al (2019)	Journal article	New Zealand	To compare the communication and practice experiences of migrant nurses in geographically distant, culturally dissimilar countries in Eastern and Western contexts.	Explorative qualitative study	Internationally qualified nurses working in New Zealand (*n* = 36) and UAE (*n* = 20).	• Internationally qualified nurses expressed a desire to learn more about the culture of the host country to overcome cultural ambiguity that may occur while practicing in a foreign cultural context.
A. Clubb (2022)	Doctoral dissertation	New Zealand	To explore internationally qualified nurses’ perceptions of their experiences on competency assessment programs (CAP) courses offered in Aotearoa. Relevant research questions: • How does your professional practice in respect to applying cultural care delivery differ now from that in your country of origin? • How did the cultural safety component in the CAP course prepare you for working in New Zealand?	Focused ethnography	Internationally qualified nurses (*n* = 12) from the Philippines and India who were 2 years post nursing registration in Aotearoa	• Internationally qualified nurses were introduced to the principles of te Triti Waitangi as a part of the competency assessment program. • The participants acknowledged the need for being sensitive to the culture in Aotearoa. • But the participants showed a lack of in-depth understanding in relation to applying the principles to overall healthcare provision as a registered nurse. The translation of their understanding was limited to task focused activities. • The participants being from multicultural background (in their home country) perceived they were prepared to be culturally safe practitioners; Conflation of cultural safety and principles of te Triti Waitangi.

### Data Analysis

Analysis of the data followed an inductive approach. Study characteristics were mapped in line with the JBI’s recommended principles of extraction, presentation and analysis, which supported the identification of key findings and themes (Pollock et al., 2023). From this approach, categories were identified as worthy of narrative discussion in line with the objectives of the review.

## Results

The initial database search yielded 574 papers, and an additional nine papers were identified from the gray literature. After the title and abstract screening, 51 papers were included for full-text review. In the end, a total of three studies were included in the final review (see Figure 1).

## Characteristics of the Included Papers

Of the three studies included, two were from New Zealand (Brunton et al., 2019; Clubb, 2022) and one from Canada (Baxter, 2017). There were no reported studies from Australia. Out of the three, two were doctoral dissertations (Baxter, 2017; Clubb, 2022), and one was a peer-reviewed journal article. Two studies followed a qualitative approach, whereas one was a mixed-method study. The participants included in the studies ranged from *n* = 10 to 36; IQNs from India and the Philippines formed the majority of the participants in all three studies (Table 2).

Baxter (2017) aimed to explore the integration of the IQNs from the Philippines into the nursing workforce of Canada, and the aim of Brunton et al. (2019) was to compare the communication and practice experiences of migrant nurses in Eastern (UAE) and Western contexts (New Zealand). Clubb (2022) aimed to explore IQNs’ perceptions of their experiences of the Competency Assessment Program (CAP) courses in Aotearoa (the Māori name for New Zealand), focusing specifically on their understanding and application of the principles of cultural safety in the context of Aotearoa culture. (Table 2).

## Knowledge and Practices of Cultural Safety

### Knowledge of Cultural Safety

Three studies highlighted that country-specific knowledge of cultural safety was limited among the IQNs. Thus, The IQNs expressed a desire to learn more about the culture specific to the Indigenous Peoples of the host country to avoid cultural ambiguity (Brunton et al., 2019). Some participants’ understanding of cultural safety was conflated with their prior understanding of multiculturalism (Clubb, 2022).

Two studies (Clubb, 2022; Baxter, 2017) identified that the formal knowledge provided as a part of pre-employment or CAPs helped facilitate the IQNs understanding of the health care system and culturally safe practices when caring for Indigenous Peoples in the host countries.

### Challenges in Translating the Knowledge to Everyday Practice

Furthermore, IQNs in one study stressed that there was a lack of in-depth understanding concerning transferring the principles of Te Triti o Waitangi, which promote the rights of self-determination and equity for Māori, to the provision of culturally safe care to Māori patients as a registered nurse (Clubb, 2022).

## Discussion

This scoping review aimed to identify and map existing evidence on knowledge and practices of cultural safety among IQNs working in Australia, New Zealand, and Canada. Our review highlighted a dearth of evidence in this field, even though IQNs comprise a significant portion of the nursing workforce in each of these countries.

Cultural safety is embedded in the Code of Conduct and professional standards for nurses in Australia, Canada, and New Zealand (British Columbia College of Nurses and Midwives, 2022; College of Nurses of Ontario, 2024; Nursing and Midwifery Board AHPRA, 2023; Nursing Council of New Zealand, 2012). These codes define the professional responsibilities of nurses and provide ethical guidelines for nursing practice. Despite this expectation that all nurses are equally equipped to provide culturally safe practice in line with these codes, our review revealed that there is a paucity of published evidence regarding cultural safety related to the IQN cohort. Our research shows that all three countries include education on cultural safety, either conducted by nursing/registration regulatory bodies or as a part of workplace induction or competency assessment, however, there is no standardized national requirement for how often these sessions must occur. This gap is seen not only for IQNs but, in fact, for all practising nurses. For example, in Australia, as a part of the health care facility orientation process, the IQNs are mandated to complete an online module related to the health of Aboriginal and Torres Strait Islander Peoples within 6 months of first registration. The module includes information about the historical factors that continue to influence the health of Aboriginal and Torres Strait Islander Peoples, the health inequity that they experience and the need for culturally safe care. In addition, the IQNs also attend cultural safety workshops if employed in public health care organizations. However, since the timing of this orientation occurs during the initial work onboarding stage, where the IQNs experience multiple adaptation and integration challenges (Dahl et al., 2022; Pung & Goh, 2017; Rajpoot et al., 2024), it is unclear how effectively this knowledge is retained and translated into practice. Since there is no standardized national requirement, the content and timing are not regulated across the health care organizations. Furthermore, clear policies for updating this knowledge are lacking in all public and private health care organizations in Australia. Since culturally safe practices require ongoing self-reflection of knowledge, skills, attitudes, and power differentials (Simmonds et al., 2023), organizational policies to support lifelong learning must be implemented to support IQNs to embrace culturally safe practices (Almansour et al., 2022; Kamau et al., 2023). Limiting cultural safety education to a one-time onboarding event may only promote cultural tokenism and fail to actively challenge existing racism and promote health equity for Indigenous Peoples (Tremblay et al., 2023).

Our review revealed a lack of cultural safety knowledge among the participants. Specifically, the participants struggled to apply their limited knowledge of cultural safety gained from pre-employment programs, in their overall role as a registered nurse. This is also in line with findings reported from nurses and midwives working in rural and remote settings in Australia (Withall et al., 2021). Research supports cultural safety training and resources be developed in collaboration with the Indigenous Communities, including the Elders and Knowledge Keepers. Such education delivered in a face-to-face mode will provide more opportunities to develop trust and promote accountability for nurses to instill the learning into their everyday practice (Hardy et al., 2023; Webb et al., 2023; Withall et al., 2021). In addition, a multi-layered approach is recommended which addresses the organizational and structural level barriers to implementing effective and collaborative education, as well as commitment from individual health care providers including nurses (Wilson et al., 2022).

Promoting positive health outcomes for Indigenous Peoples requires nurses to critically reflect on their privileges, the power dynamics, positionality and internal biases. Engaging in ongoing critical reflection is facilitated by historical knowledge of socio-political invasion and intergenerational trauma that Indigenous Peoples have experienced and continue to experience. There is a professional imperative for IQNs to be provided opportunities to both gain knowledge of cultural safety, and to develop the skills and confidence to apply culturally safe practices when they are caring for Indigenous People. Our review provides several recommendations for research, practice and policy as follows:

### Research

Our review underscores the need for further research to examine existing knowledge about cultural safety understanding among IQNs, and how this knowledge is translated to mitigate health inequities affecting Indigenous Peoples. Furthermore, the findings from this review can inform future research to explore appropriate theoretical frameworks and culturally safe training programs, including the longitudinal impacts of such programs on nursing practice and health outcomes for Indigenous Peoples.

### Practice

To ensure culturally safe care as determined by the Indigenous Peoples is being provided, evidence-based best practice guidelines should be developed and implemented to support IQNs’ continuous knowledge and practice development. Resources must be developed collaboratively with Indigenous Peoples and provide opportunities for engagement with Indigenous cultures beyond online on-boarding, or orientation packages. In addition, a practice environment that fosters ongoing reflection and education of culturally safe practice will support all nurses to continue to engage in the development of their own cultural capabilities.

### Policy

Policies that support lifelong learning and critical self-reflection for culturally safe practices must be encouraged as a standard practice across all private and public health care organizations. Furthermore, nursing regulatory bodies across Australia, New Zealand, and Canada could introduce policies mandating cultural safety training as a requirement for registration renewal or as a specified component of continuous professional development.

## Strengths and Limitations

This scoping review attempted to map the extent and type of evidence related to knowledge and skills of cultural safety among IQNs working in Australia, Canada, and New Zealand. The review highlighted a significant dearth of evidence in this area, and provides recommendations for future research, practice and policy improvement.

There are some limitations to this review. Of the reviewed papers, only one was a peer-reviewed article. Furthermore, although our review focused on three countries, there were no studies available from Australia. In addition, although there are Indigenous Peoples in other countries including the United States, these were excluded from our review due to differences in the health care system and the focus on cultural safety education in nursing curricula compared with the three countries included in our study.

## Conclusion

Culturally safe practices are critical to address the ongoing health inequities experienced by Indigenous Peoples while accessing health care. Our review identified that knowledge of culturally safe practices among the IQNs is not well supported with the current educational opportunities, despite professional imperatives to ensure culturally safe care for Indigenous People. It is also evident that IQNs face challenges translating the limited knowledge into the delivery of culturally safe health care practice. Further research is urgently needed to generate evidence in this area, which will guide in planning appropriate education and policies to ensure safe, accessible, equitable, and racism-free health care for Indigenous Peoples.

## Supplemental Material

sj-docx-1-tcn-10.1177_10436596251353518 – Supplemental material for Cultural Safety Knowledge and Practices Among Internationally Qualified Nurses Caring for Indigenous Peoples in Australia, New Zealand and Canada: A Scoping Review Supplemental material, sj-docx-1-tcn-10.1177_10436596251353518 for Cultural Safety Knowledge and Practices Among Internationally Qualified Nurses Caring for Indigenous Peoples in Australia, New Zealand and Canada: A Scoping Review by Pratibha Bhandari, Ling Zeng, Anne-Marie Eades, Danielle Manton, Annie Hepworth, Carolyn Antoniou, Elaine Correia Moll, Jack Cornish and Suzanne Sheppard-Law in Journal of Transcultural Nursing
