# Supplementary material for: Cultural Safety Knowledge and Practices Among Internationally Qualified Nurses Caring for Indigenous Peoples in Australia, New Zealand and Canada: A Scoping Review
Source: J Transcult Nurs. 2025 Jul 22;36(6):721–9. doi: 10.1177/10436596251353518 (PMC12521763; doi:10.1177/10436596251353518)
Supplement: sj-docx-1-tcn-10.1177_10436596251353518 – Supplemental material for Cultural Safety Knowledge and Practices Among Internationally Qualified Nurses Caring for Indigenous Peoples in Australia, New Zealand and Canada: A Scoping Review [file sj-docx-1-tcn-10.1177_10436596251353518.docx]

**Supplementary table 1: Search strategies last access 24/07/2024)**

| **Database** | **Search strategy** | **Filters** | **Result** |
| --- | --- | --- | --- |
| Scopus | ( TITLE-ABS-KEY ( "Internationally qualified nurs*" OR "Overseas qualified nurs*" OR "Internationally trained nurs*" OR "Overseas trained nurs*" OR "Internationally educated nurs*" OR "IEN" OR "International nurs*" OR "Migrant nurs*" OR "Foreign-educated nurs*" ) AND TITLE-ABS-KEY ( australia* OR canad* OR "New Zealand" ) AND TITLE-ABS-KEY ( cultural* ) ) | English | 109 |
| Web of Science | “Internationally qualified nurs*” OR “Overseas qualified nurs*” OR “Internationally trained nurs*” OR “Overseas trained nurs*” OR “Internationally educated nurs*” OR “IEN” OR “International nurs*” OR “Migrant nurs*” OR “Foreign-educated nurs*” (Topic) and Australia* OR Canad* OR “New Zealand” (Topic) and cultural* (Topic) and Preprint Citation Index (Exclude – Database) | English | 107 |
| Medline (Ovid) | (("Internationally qualified nurs*" or "Overseas qualified nurs*" or "Internationally trained nurs*" or "Overseas trained nurs*" or "Internationally educated nurs*" or "IEN" or "International nurs*" or "Migrant nurs*" or "Foreign-educated nurs*").mp.) OR Nurses, International/ AND (Australia* or Canad* or "New Zealand").mp. AND (cultural*.mp. OR Cultural Competency/ or  Culturally Competent Care/ or Cultural Diversity/ or Transcultural Nursing/ or Clinical Competence/) | English | 95 |
| Embase | (("Internationally qualified nurs*" or "Overseas qualified nurs*" or "Internationally trained nurs*" or "Overseas trained nurs*" or "Internationally educated nurs*" or "IEN" or "International nurs*" or "Migrant nurs*" or "Foreign-educated nurs*").mp.or foreign nurse/) AND (Australia* or Canad* or "New Zealand").mp. AND (Cultural*.mp. or cultural competence/ or transcultural care/ or cultural diversity/ or clinical competence/) | English | 100 |
| CINAHL | (“Internationally qualified nurs*” OR “Overseas qualified nurs*” OR “Internationally trained nurs*” OR “Overseas trained nurs*” OR “Internationally educated nurs*” OR “IEN” OR “International nurs*” OR “Migrant nurs*” OR “Foreign-educated nurs*” OR (MH "Foreign Nurses") ) AND (Australia* OR Canad* OR “New Zealand”) AND ( Cultural* OR (MH "Transcultural Care") OR (MH "Clinical Competence") OR (MH "Cultural Safety") OR (MH "Cultural Diversity") OR (MH "Cultural Competence") ) | English | 163 |
